# The spiral evolution of techniques for the laboratory diagnosis of microbial infections

**DOI:** 10.3389/fcimb.2026.1922970

**Published:** 2026-08-27

**Authors:** Yi-Wei Tang, Nahed Ismail, Rodolfo García-Contreras

**Affiliations:** 1 School of Public Health, Chongqing Medical University, Chongqing, China; 2 Department of Pathology, University of Illinois at Chicago, Chicago, IL, United States; 3 Department of Microbiology and Parasitology, Faculty of Medicine, Universidad Nacional Autónoma de México, Mexico City, Mexico

**Keywords:** clinical microbiology, genotype, molecular phenotype, phenotype, spiral evolution

## Abstract

The laboratory diagnosis of microbial infections has undergone a profound transformation over the past century, evolving from classical phenotypic methods to molecular genetic approaches and now toward ultra-sensitive molecular phenotyping. While this progression is often described as linear, a closer examination reveals a cyclical pattern: an initial reliance on observable biological function, followed by a shift toward genetic abstraction, and, more recently, a return to phenotypic measurement at unprecedented molecular resolution. This “diagnostic circle” reflects a deeper epistemological trajectory shared across multiple scientific disciplines, wherein knowledge alternates between direct observation and abstraction before converging into integrative, high-resolution frameworks. In this Perspective, we trace the evolution of diagnostic microbiology through three major phases - classical phenotypic methods, molecular/genetic diagnostics, and emerging ultra-sensitive phenotypic technologies - and argue that the field is entering a new era in which functional biology can be measured directly with molecular precision. We propose that this transition represents not merely technological progress but a conceptual shift toward restoring biological context, with significant implications for clinical decision-making, antimicrobial stewardship, and the future of precision infectious disease diagnostics.

## Introduction

The diagnosis of microbial infections lies at the intersection of biology, technology, and clinical decision-making (Patel and Fang, 2018). For over a century, laboratory microbiology has provided the evidentiary backbone for identifying pathogens, guiding therapy, and monitoring disease progression (Caliendo et al., 2013; Schmitz et al., 2022; Wang et al., 2022). The trajectory of diagnostic innovation is often portrayed as a straightforward advancement from slower, less sensitive methods to faster and more sensitive and precise ones. However, such a linear narrative obscure a more nuanced reality.

A broader view reveals that diagnostic microbiology has followed a cyclical path. Early methods emphasized phenotype—the observable characteristics of microorganisms, including growth, morphology, and biochemical traits. The advent of molecular biology shifted the focus toward genotype, enabling detection of microbial nucleic acids with extraordinary sensitivity and specificity. Today, emerging technologies are enabling a return to phenotypic measurement, but at a fundamentally different scale: the molecular and even single-cell level.

**Figure 1:**
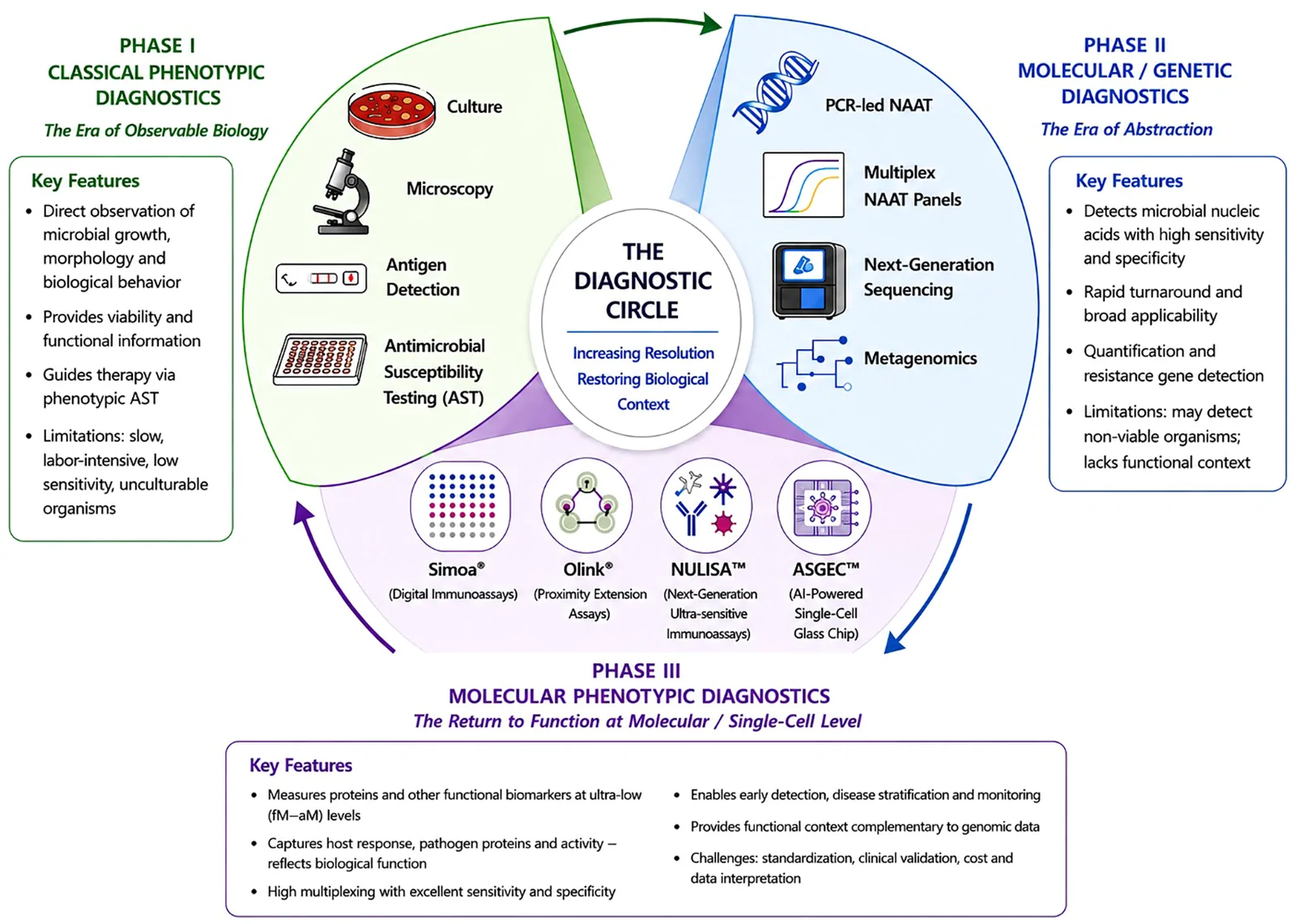
The spiral evolution of techniques for the laboratory diagnosis of microbial infections. Laboratory diagnostics for microbial infections have evolved through three major phases: Phase I, classical phenotypic methods based on observable biology: Phase ll, molecular/genetic approaches focused on nucleic acid detection and characterization; and Phase III, molecular phenotypic methods that return to measuring biological function at molecular and single-cell levels. This cycle reflects increasing resolution and the restoration of biological context.

This cycle—from phenotype to genotype and back to phenotype—mirrors patterns observed in other scientific disciplines, including biology, physics, and philosophy. Each phase addresses the limitations of the previous one while introducing new constraints. Understanding this cycle is not merely of historical interest; it provides a conceptual framework for anticipating the next generation of diagnostic tools and their clinical impact (**Figure 1**).

This Perspective is intended for readers with a strong background in clinical microbiology and laboratory diagnostics. Its primary objective is to highlight conceptual advances and emerging technologies that are shaping the future of infectious disease diagnostics rather than to provide a comprehensive technical review of individual methodologies. Detailed discussions of the analytical principles, technical implementation, validation, and mechanistic aspects of specific diagnostic platforms are beyond the scope of this article and are available in specialized reviews.

## Phase I: classical phenotypic diagnostics — the era of observable biology

### Foundations of phenotypic microbiology

The origins of diagnostic microbiology are rooted in the ability to observe and cultivate microorganisms (Baron et al., 2013; Leo et al., 2020; Schmitz et al., 2022). Techniques such as culture, microscopy, biochemical activity, and antigen detection formed the cornerstone of laboratory diagnosis throughout the 20th century. These methods were grounded in the direct observation of biological phenomena: microbial growth, colony morphology, staining characteristics, and enzymatic reaction.

Culture-based methods provided a powerful means of isolating pathogens and assessing their functional properties. The ability to grow organisms *in vitro* enabled not only microbial identification but also antimicrobial susceptibility testing (AST), which remains a critical component of clinical care. Phenotypic assays thus offered a comprehensive view of microbial behavior, integrating viability, metabolic activity, and drug response.

### Strengths and limitations

The strength of phenotypic methods lies in their biological relevance. A positive culture result confirms the presence of viable organisms capable of replication and causing disease, while susceptibility testing directly informs therapeutic choices. These methods inherently capture the complexity of microbial physiology and host–pathogen interactions.

However, phenotypic diagnostics are constrained by several limitations. They are often slow with delayed turnaround times (TAT), requiring hours to days for results. For example, phenotypic antimicrobial susceptibility testing provides functionally relevant evidence by directly measuring the response of a viable pathogen to antimicrobial exposure. Nevertheless, the methods depend on organism recovery and measurable growth, frequently delaying actionable results until after empirical therapy has been initiated. This delay creates a clinical tension: broad-spectrum treatment may be necessary to avoid inadequate initial coverage, but continued empirical therapy increases toxicity, microbiome disruption and selection pressure. Moreover, *in vitro* susceptibility does not fully reproduce drug exposure, host immunity, infection-site conditions, biofilms, heteroresistance, tolerance or persistence, and MIC measurements have inherent biological and methodological variability (Gajic et al., 2022).

Many pathogens are fastidious or unculturable, leading to false negatives. Sensitivity may be limited, particularly in cases of low microbial loads or after antimicrobial exposure (AlJerf et al., 2026). Moreover, majority of phenotypic assays typically provide limited quantitative information and may lack specificity in the context of polymicrobial infections. Another key limitation of phenotypic testing is the requirement for highly trained laboratory personnel or technologists, who may not be readily available in all settings. This is further compounded by a shortage of microbiology staff, and the extensive training needed to achieve and maintain competency, which can be challenging for many laboratories. These challenges set the stage for a paradigm shift toward molecular diagnostics.

## Phase II: molecular and genetic diagnostics — the era of abstraction

### The rise of nucleic acid-based methods

The introduction of polymerase chain reaction (PCR) and related nucleic acid amplification technologies revolutionized diagnostic microbiology (Mullis and Faloona, 1987; Tang et al., 1997; Gu et al., 2019). For the first time, pathogens could be detected based on their genetic material, independent of viability or cultivability (Tang et al., 1997). This transition marked a shift from observing what microorganisms *do* at phenotype level to identifying what they *are* at the molecular level.

Molecular diagnostics rapidly expanded to include not only qualitative PCR detection of an individual pathogen in human specimens, but also quantitative PCR (qPCR), multiplex panels, and next-generation sequencing (NGS) (Ramanan et al., 2017; Schmitz et al., 2022). These tools enabled highly sensitive and specific detection of pathogens, often within hours. They also facilitated the identification of antimicrobial resistance genes and the characterization of microbial communities through metagenomic approach (Gu et al., 2019; Schmitz et al., 2022; Gomes et al., 2025).

### Strength and limitations of the molecular paradigm

Molecular diagnostics offer several key advantages. They are rapid, sensitive, and broadly applicable, capable of detecting a wide range of pathogens, including viruses and unculturable organisms. Quantitative assays provide measures of microbial load, which can be useful for monitoring disease progression and treatment response.

Importantly, molecular methods have transformed the management of infectious diseases, particularly in the diagnosis of respiratory infections, blood stream infections, and viral illness. The advent of molecular biology also resulted in rapid identification of antimicrobial resistance by detecting resistance-related genes and mutations. Multiplex syndromic panels enable the simultaneous detection of a broad range of pathogens, and in some cases, key antimicrobial resistance markers directly from clinical specimens, improving diagnostic yield and reducing time to pathogen detection. These technical advances support earlier therapeutic decision-making and antimicrobial stewardship.

Despite their transformative impact, genetic techniques—including NGS and metagenomic approaches—introduce a degree of biological abstraction because they characterize microbial genomes rather than directly assessing microbial phenotype or function. Detection of nucleic acids does not necessarily indicate the presence of viable or active organisms. Colonized or residual pathogen nucleic acids may persist, even after infection has been resolved, leading to potential overdiagnosis (Polage et al., 2015). Furthermore, molecular assays often lack functional context. The presence of a resistance gene does not guarantee phenotypic resistance, and the absence of such a gene does not preclude it. Similarly, pathogen detection does not directly inform host response or disease severity.

In this sense, molecular diagnostics trade biological richness for analytical precision. They excel at answering the question “Is the genetic material present?” but are less equipped to address “Is the organism active, and what is it doing?” These limitations have driven the search for methods that can combine sensitivity and specificity with functional relevance.

## Phase III: ultra-sensitive molecular phenotyping — the return to function

### Emergence of high-sensitivity protein and functional assays

**Figure 2:**
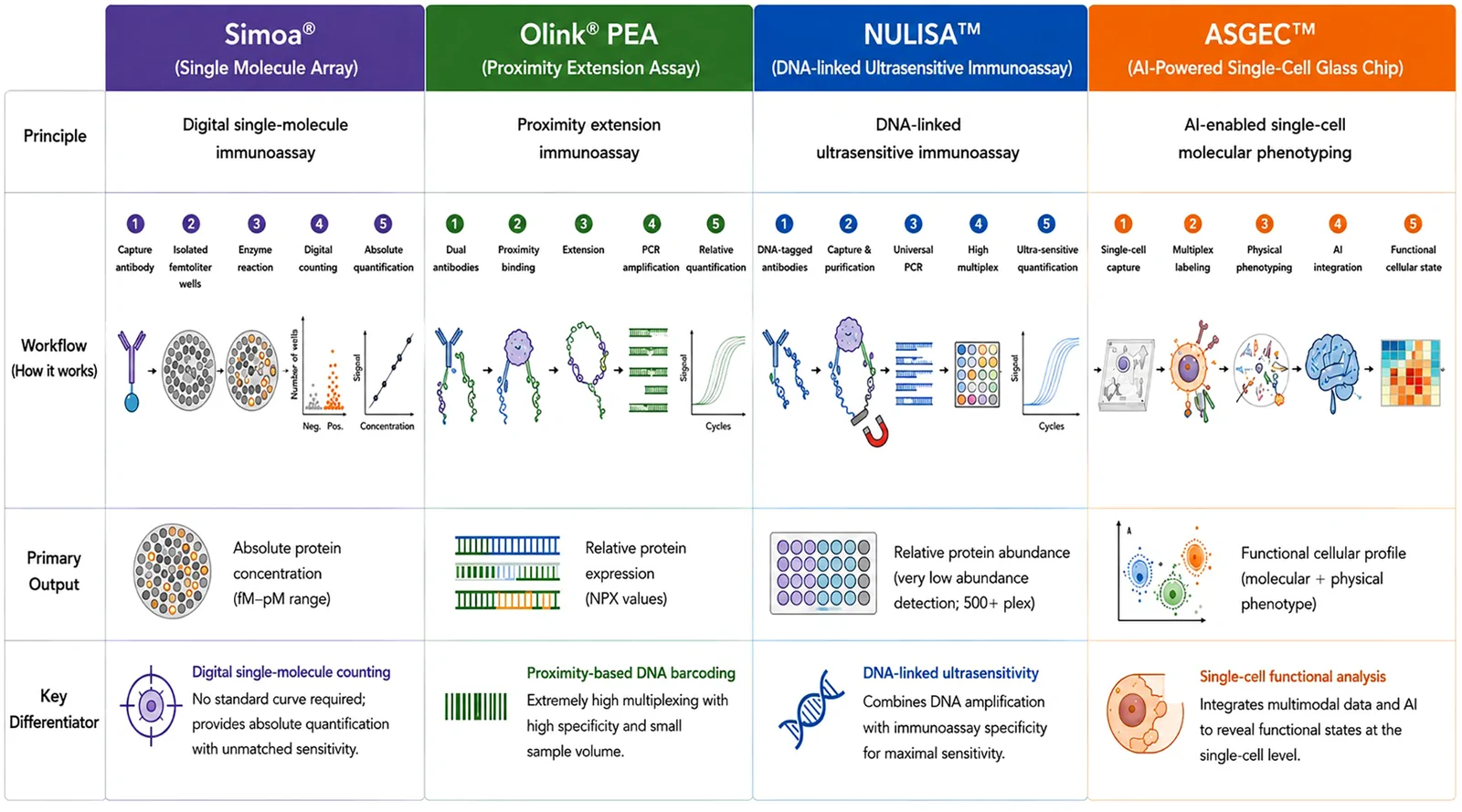
Principles of four next generation molecular phenotyping technologies. The figure illustrates how each assay works from sample to digital quantification. Sinoa performs digital single-molecule immunoassay in femtoliter wells enabling absolute protein quantification. Oink PEA uses proximity extension and DNA barcoding for highly multiplexed relative quantification. NULISA employs DNA inked immunoassays with universal PCR for ultra-sensitive, high-multiplex protein detection. ASGEC integrates single-cell capture, multiplex labeling, microfluidics, imaging and AI analysis to define functional cellular states.

Recent advances in analytical technologies are enabling a new generation of diagnostic tools that measure biological function at the molecular level. Platforms such as digital immunoassays, proximity extension assays, and aptamer-based proteomics can detect proteins and other biomarkers at femtomolar (10^-^¹^5^ M) to sub-femtomolar concentrations, which is approximately 100–1000 times more sensitive than conventional ELISA. Examples include: 1. Single-molecule array (Simoa) technology, which enables digital quantification of proteins at extremely low abundance (Rissin et al., 2010). 2. Proximity extension assays (PEA), exemplified by Olink, which allow multiplexed protein detection with high specificity (Assarsson et al., 2014). 3. Emerging platforms such as NULISA, which aim to further improve sensitivity and scalability (Feng et al., 2023). These technologies represent a return to phenotypic measurement, but with a level of sensitivity and multiplexing that was previously unattainable (**Figure 2**; **Table 1**).

**Table 1:** Four ultra-sensitive proteomic technology comparison.

Parameters	Simoa	Olink	NULISA	ASGEC
Core principle	Digital single-molecule immunoassay	Proximity Extension Assay (PEA)	Antibody-DNA barcode immunoassay	Digital single-molecule immunoassay
Quantitation type	Absolute quantitation with sampling variation (pg/mL)	Relative NPX units (normalized expression)	Relative abundance units (platform-specific)	Absolute quantitation (pg/mL)
Sensitivity	Ultra-high (fg/mL to low pg/mL)	Very high (fM range)	Claimed ultra-high (sub-fM/attomolar for some analytes)	Ultra-high (fg/mL to low pg/mL)
Specificity mechanism	Traditional dual-antibody sandwich specificity	Dual-antibody coincidence detection	Dual-antibody + low-background molecular architecture	Digitizing immune responses and single-cell physical properties
Multiplexing capability	Low–moderate (typically 1–10 plex; some higher for mats emerging)	Moderate–high (~92–1,500 proteins)	Moderate–high and expanding	Low–moderate (typically 1–10 plex; some higher for mats emerging)
Dynamic range	Very wide	Wide	Expected wide	Very wide
Throughput	Moderate–high automation	Very high cohort scalability	Still scaling operationally	Very high cohort scalability
Main limitation	Limited multiplex scale	Relative rather than absolute quantitation	Less validation and standardization so far	Eary development stage
Application status	IVD device available	Primarily RUO	RUO	R&D stage

Single-molecule array (Simoa) technology represents one of the earliest and most influential ultra-sensitive protein detection platforms (Rissin et al., 2010). By partitioning individual immunocomplexes into thousands of femtoliter-sized reaction wells and digitally counting enzyme-labeled events, Simoa converts conventional analog immunoassays into a digital readout capable of detecting proteins at femtomolar to sub-femtomolar concentrations. This dramatic gain in analytical sensitivity enables quantification of extremely low-abundance biomarkers that were previously undetectable in blood, including cytokines, neurodegenerative disease markers, tumor-associated proteins, and early infection biomarkers. This technology effectively bridges traditional immunoassays and single-molecule detection, providing high precision, broad dynamic range, and growing multiplexing capabilities for translational research and clinical diagnostics. In March 2024, the US Food and Drug Administration granted Breakthrough Device Designation to Quanterix’s Simoa p-Tau217 assay, which successfully addressed the limitations of traditional phenotypic methods including delayed turnaround times and limited sensitivity (Palmqvist et al., 2020).

Proximity Extension Assay (PEA), commercialized by Olink, combines dual-antibody recognition with DNA-based signal generation to achieve exceptional specificity and multiplexing (Assarsson et al., 2014). Two antibodies labeled with complementary oligonucleotides must bind near the same target protein before a unique DNA barcode is generated and amplified by PCR. This proximity-dependent mechanism substantially reduces nonspecific background signals ensuring high specificity of the assay, while preserving high analytical sensitivity. Modern PEA platforms can simultaneously quantify hundreds to thousands of both high and low abundant proteins from minimal sample volumes, making them particularly valuable for biomarker discovery, systems biology, longitudinal cohort studies, infectious diseases, oncology, and precision medicine applications. Importantly, PEA provides a scalable framework for high-dimensional proteomic profiling while maintaining strong reproducibility across large clinical studies.

NULISA (Nucleic Acid-Linked Immuno-Sandwich Assay) represents a next-generation evolution of proximity-based immunoassays (Feng et al., 2023). Building upon concepts established by proximity ligation assay (PLA) and PEA, NULISA incorporates innovative background-suppression strategies, including dual capture-and-release purification and DNA-barcoded reporter systems, enabling attomolar-level protein detection with high multiplexing capacity. Recent studies demonstrated that NULISA can improve analytical sensitivity by several orders of magnitude compared with conventional proximity assays while maintaining broad dynamic range and compatibility with next-generation sequencing readouts. These capabilities allow simultaneous measurement of large panels of ultra-low-abundance cytokines, chemokines, host-response biomarkers, and disease-associated proteins, providing an unprecedented view of biological activity in infectious diseases, autoimmune disorders, cancer, and other complex conditions. As automation and clinical validation advance, NULISA may further accelerate the transition from genomic characterization to functional molecular phenotyping.

The artificial intelligence (AI)-powered Single-Cell Glass-Based Electronic Chip (ASGEC) represents an emerging platform that extends molecular phenotyping beyond soluble biomarker quantification to direct characterization of individual cells. Built upon a glass-based microfluidic architecture, ASGEC integrates automated sample processing, multiplex immunofluorescent labeling, high-speed imaging, microfluidic flow dynamics, and artificial intelligence–driven analytics within a single platform. Unlike conventional proteomic technologies that primarily measure circulating proteins, ASGEC simultaneously captures both the biochemical fingerprint (e.g., CD4, CD8, CD19, p-tau217, and other disease-associated markers) and the physical fingerprint (e.g., cell size, deformability, mechanical properties, and flow behavior) of individual cells. By applying deep-learning algorithms to integrate these multidimensional datasets, the platform generates functional cellular phenotypes and predictive biomarkers such as immune fitness indices, neurodegenerative disease risk signatures, and advanced leukocyte classifications (Tang et al, unpublished data). This approach enables the creation of dynamic digital cellular profiles that bridge morphology, molecular biology, and function. As a result, ASGEC exemplifies the next evolutionary step in diagnostic microbiology and precision medicine, where cellular function can be quantified, digitized, and interpreted in real time through AI-assisted analysis.

### Reconstructing phenotypes at molecular resolution

Unlike classical phenotypic methods, which rely on macroscopic observations such as growth, these new approaches measure molecular signatures of biological activity. They capture aspects of host response (e.g., cytokines, acute-phase proteins), pathogen-derived proteins, and other functional biomarkers (Sweeney et al., 2016; Hanson and Tsalik, 2025). In effect, they allow the reconstruction of phenotypes from their molecular components. This represents a synthesis of the strengths of both earlier phases: the biological relevance of phenotypic assays and the sensitivity and speed of molecular methods.

### Clinical implications

Ultra-sensitive phenotyping has the potential to transform infectious disease diagnostics in several ways: 1. Early detection of infection before overt clinical symptoms. 2. Differentiation between infection and colonization based on host response. 3. Improved antimicrobial stewardship through better assessment of disease activity. 3. Monitoring of treatment response with dynamic biomarker profiles. However, challenges remain, including standardization, cost, and the need for robust clinical validation and quality assurance. To date, the four ultra-sensitive molecular phenotypic techniques can detect multiple biomarkers simultaneously, making them ideal for integration with AI and machine learning for future diagnostic applications.

## The diagnostic circle: a conceptual framework

### From phenotype to genotype and back

The evolution of diagnostic microbiology can be conceptualized as a circle rather than a linear progression. The field began with phenotypic observation, moved toward genetic abstraction, and is now returning to phenotype—albeit at a higher level of resolution.

This pattern can be summarized as: 1. Phenotypic phase: Direct observation of biological function. 2. Genotypic phase: Indirect detection via molecular signatures. 3. Molecular phenotypic phase: Direct measurement of function at molecular scale. Each phase addresses the limitations of the previous one while introducing new capabilities.

### Parallels in other disciplines

This cyclical pattern seems not unique to microbiology. In philosophy, one observes progression from materialism (focus on observable reality) to idealism (emphasis on abstract concepts) and then to dialectical materialism, which integrates both. In biology, the field has moved from morphology to genomics and now to systems biology and multi-omics. Similar patterns are observed in physics, chemistry, and neuroscience (**Table 2**).

**Table 2:** Parallels across disciplines: a common pattern of knowledge evolution.

Evolution phase	Philosophy	Chemistry	Biology	Clinical and diagnostic microbiology and immunology
Phase I (Phenotype/ Observation)	Materialism (Empiricism)	Wet Chemistry (Observation)	Morphology (Phenotype)	Phenotype
Phase II (Genotype/ Abstraction)	Idealism (Rationalism)	Quantum Chemistry/Computational Models	Molecular Biology/Genomics Genes & Molecules)	Genotype
Phase III (Molecular Phenotype Synthesis)	Dialectical Materialism (Synthesis/Praxis)	Operando Chemistry (Real-time Molecular Observation)	Systems Biology/Single-cell Multi-omics (Function in Context)	Molecular phenotype

These parallels suggest that the diagnostic circle reflects a broader epistemological dynamic: the interplay between observation and abstraction in the pursuit of knowledge.

## Implications for the future

### Toward integrated diagnostics

The future of infectious disease diagnostics is likely to involve integration across modalities. Rather than replacing molecular methods, ultra-sensitive phenotyping will complement them, providing a more complete picture of infection. For example, combining pathogen detection with host-response biomarkers may enable more accurate diagnosis and risk stratification. Multi-omics approaches that integrate genomics, proteomics, and metabolomics could further enhance diagnostic precision.

### Restoring biological context

A key theme in the diagnostic circle is the restoration of biological context. While molecular methods excel at detection, they often lack information about functional relevance. Emerging phenotypic technologies aim to fill this gap by capturing dynamic biological processes. This shift has important implications for clinical decision-making. Diagnostics that reflect real-time biological activity may enable more personalized and adaptive treatment strategies.

### Challenges and considerations

Despite their promise, new technologies must overcome several hurdles including (1) standardization and reproducibility, (2) clinical validation and regulatory approval, (3) cost and accessibility, and (4) data interpretation and integration. Addressing these challenges will be essential for translating technological advances into clinical benefits. In addition, how will the increased sensitivity of these technologies influence our detection and characterization of resistant microbial populations awaits to be answered.

## Conclusion

The history of diagnostic microbiology is not a simple story of linear progress but a spiral evolution. From the direct observation of microbial behavior to the abstraction of genetic information and now to the reconstruction of phenotype at molecular resolution, each phase reflects a different way of understanding biological reality.

Recognizing this “diagnostic circle” provides a valuable framework for interpreting current trends and anticipating future developments. As the field moves toward ultra-sensitive phenotyping, it is poised to reconcile the strengths of past approaches, combining sensitivity with biological relevance.

Ultimately, the goal of diagnostic innovation is not merely to detect pathogens but to understand their role in disease. By returning to phenotype—now enriched by molecular precision—diagnostic microbiology may achieve a more complete and clinically meaningful representation of infection. As this paradigm continues to evolve, it will be essential to ensure that these emerging technologies are developed, validated, and implemented in ways that preserve biological context, enhance clinical relevance, and ultimately improve patient care.

## Data Availability

The original contributions presented in the study are included in the article/supplementary material. Further inquiries can be directed to the corresponding author.
